# Particulate Matter Pollution and Population Exposure Assessment over Mainland China in 2010 with Remote Sensing

**DOI:** 10.3390/ijerph110505241

**Published:** 2014-05-14

**Authors:** Ling Yao, Ning Lu

**Affiliations:** State Key Laboratory of Resources and Environmental Information System, Institute of Geographic Sciences and Natural Resources Research, Chinese Academy of Sciences, Beijing 100101, China; E-Mail: yaoling@lreis.ac.cn

**Keywords:** particulate matter pollution, population exposure, air quality assessment, remote sensing

## Abstract

The public is increasingly concerned about particulate matter pollution caused by respirable suspended particles (PM_10_) and fine particles (PM_2.5_). In this paper, PM_10_ and PM_2.5_ concentration are estimated with remote sensing and individual air quality indexes of PM_10_ and PM_2.5_ (IPM_10_ and IPM_2.5_) over mainland China in 2010 are calculated. We find that China suffered more serious PM_2.5_ than PM_10_ pollution in 2010, and they presented a spatial differentiation. Consequently, a particulate-based air quality index (PAQI) based on a weighting method is proposed to provide a more objective assessment of the particulate pollution. The study demonstrates that, in 2010, most of mainland China faced a lightly polluted situation in PAQI case; there were three areas obviously under moderate pollution (Hubei, Sichuan-Chongqing border region and Ningxia-Inner Mongolia border region). Simultaneously, two indicators are calculated with the combination of population density gridded data to reveal Chinese population exposure to PM_2.5_. Comparing per capita PM_2.5_ concentration with population-weighted PM_2.5_ concentration, the former shows that the high-level regions are distributed in Guangdong, Shanghai, and Tianjin, while the latter are in Hebei, Chongqing, and Shandong. By comparison, the results demonstrate that population-weighted PM_2.5_ concentration is more in line with the actual situation.

## 1. Introduction

In recent years, inhalable particulate matter has become the primary air pollutant in China [1]. Many studies have shown that fine particulate matter concentration has a certain association with pollution-related disease mortality [2,3,4,5]. Public opinion now pays more and more attention to PM_2.5_ because of its connection to health risks [6], but an often neglected factor is coarser particles (PM_2.5_ to PM_10_) formed by dust suspensions (from road dust and dust storms) which could also have a great influence on human health.

Nowadays, air quality index (AQI), a numerical model of air quality evaluation based on the pollution standards index (PSI) proposed by the United States Environmental Protection Agency (EPA) in the 1970s, is widely used to inform the public how polluted the air is. Many countries and regions around the world employ different classification methods according to their characteristics. An individual air quality index (IAQI) is assigned to the level of each pollutant and the current AQI depends on the primary pollutant level (the highest IAQI of the 6 scores); although convenient it cannot accurately reflect the particulate pollution state. Many studies based on revised models of AQI or fuzzy set theory also face the same situation [7,8].

Furthermore, launching a particulate matter concentration monitoring network to determine the distribution situation faces phenomenally high costs [7]. The density of the particulate matter concentration ground observation network in China is relatively low, and the distribution of observation stations is uneven. Remote sensing techniques, which provide consistent measurements at broad-scale and frequent time intervals, have been increasingly used to assess surface level of particulate matter concentration at high spatial and temporal resolutions [9]. Hence, remote sensing retrieval data is introduced in this paper.

As to population exposure to particulate matters in China, some relevant research has been done in specific cities, such as Beijing [7,10,11], Shanghai [12], Wuhan [13], Lanzhou [14], and Hong Kong [15], but the spatial differentiation of population exposure to PM_2.5_ is rarely reported.

In this paper, we focus on the evaluation of atmospheric particulate matter pollution and propose a new particulate air pollution index (PAQI) which only takes PM_10_ and PM_2.5_ into account. Specifically, we use the retrieved PM_2.5_ and PM_10_ concentration over mainland China in 2010 to calculate the individual indexes of PM_2.5_ (IPM_2.5_) and PM_10_ (IPM_10_). Furthermore, the spatial distribution of PAQI over mainland China in 2010 is discussed. Meanwhile, two indicators of population exposure to PM_2.5_ are calculated and discussed.

The rest of this paper is organized as follows: in Section 2, we give a brief description of the study area, data sources, and methodologies. Then in Section 3.1 we discuss and compare the spatial distributions of IPM_2.5_, IPM_10_, and PAQI over mainland China in 2010. Section 3.2 discusses Chinese population exposure to PM_2.5_ in 2010. Finally, we summarize our work and draw some conclusions.

## 2. Materials and Methods

### 2.1. Study Area and Data Sources

In this paper, our spatial domain includes mainland China (excluding Hainan Province, which is not included in the study area due to the influence of clouds and aerosol type). Three data sources are used in the study:

(1) MODIS atmosphere aerosol product (MOD04_L2) is derived from National Aeronautics and Space Administration (NASA) at a spatial resolution of 0.1 degree, details on this data can be found elsewhere [16,17].
(2) Meteorological data (ERA-Interim), the latest global atmospheric reanalysis data produced by the European Centre for Medium-Range Weather Forecasts (ECMWF). Dee *et al.* [18] have covered the details of the ERA-Interim product.
(3) Population density gridded data derived from the Center for International Earth Science Information Network (CIESIN) contains the human population distribution. It is a gridded data product that renders global population data at the scale and extent required to demonstrate the spatial relationship of human population and the environment across the globe.

### 2.2. PM_10_ and PM_2.5_ Concentration Estimation

Gupta *et al.* have determined that the artificial neural network (ANN) algorithm can solve the PM estimation problem and there are some relevant studies about this topic [9,19,20]. In this paper, we make the following improvements to these models: the Levenberg-Marquardt (L-M) back-propagation ANN is introduced to build the particulate matter concentration estimation model in this research. First, we use stepwise regression to determine the input variables: latitude, longitude, wind speed (WS), relative humidity (RH), skin temperature (SKT), boundary layer height (HPBL), aerosol optical thickness (AOT), and single scattering albedo (SSA). Among these elements, AOT and SSA are obtained from MOD04_L2, and the other four are derived from ERA-Interim. This step can make the neural network waste less resources in extracting and fitting irrelevant features. 

**Figure 1:**
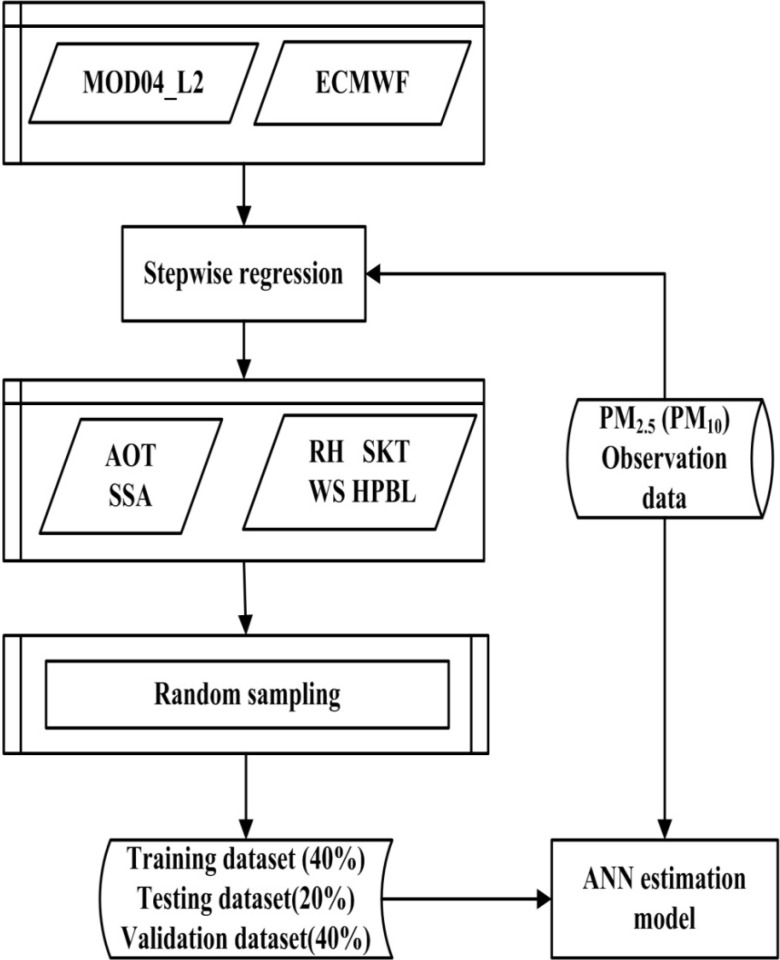
The PM concentration estimation modeling process with ANN.

The estimation modeling process is described in Figure 1. We use data of year 2008 to build the estimation model and then estimate the PM concentration in 2010. As to the ANN parameters, it has been proven that neural networks with a single hidden layer are universal approximators capable of representing any real-valued continuous function to arbitrary precision over a finite domain if enough hidden nodes are used [21]. However, networks with multiple hidden layers can sometimes perform better than single-hidden-layer networks, with fewer total nodes. In this paper, the experiment shows that over 16 hidden nodes and 9,000 runs with random sampling provided sufficient data samples for the used training data, because the satellite retrieval accuracy of PM concentration almost remained the same as the number of hidden nodes and runs increased subsequently. Consequently, the ANN estimation model is constructed with eight nodes and 16 hidden layers. The accuracy of estimation model will be discussed in Section 3.1.

### 2.3. PAQI Calculation

Though AQI is calculated on the basis of ground monitoring data, it only reports the air quality around each monitoring stations. However, satellite retrievals can exhaustively get the air quality spatial distribution, even if it’s not accurate enough. Before the PAQI calculation, IPM_2.5_, IPM_10_ are calculated with the estimation data according to the Chinese AQI standard [22]. PAQI calculation is based on a weighting method; weight values are given to IPM_2.5_ and IPM_10_, respectively, according to a certain condition [23,24], that is, individual indexes and its weight value equal PAQI. In this paper, we only consider PM_10_ and PM_2.5_ (that is, *i* = 2), however, it’s scalable if we have other PM data (PM_1_, PM_0.5_, *etc.*) in the future. First, we calculate the weighting of the *i*th individual index (Q*_i_*). Suppose:

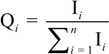
(1)
where, 0 ≤ Q*_i_* ≤ 1 (*i* = 0,1,2,…), I*_i_* is the *i*th individual air quality index. However, if we calculate PAQI with Q*_i_*, it would be not relate well with the facts under certain circumstances, for example, the dust-stormy weather, IPM_10_ is extremely high while IPM_2.5_ is relatively low. Hence, we propose the weight assignment equation (Equation 2) is:

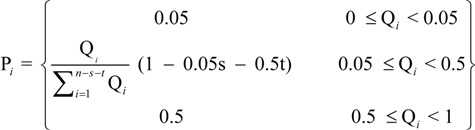
(2)
where, *s* is the number that 0≤ Q*_i_* < 0.05; *t* is the number that 0.5 < Q*_i_* ≤ 1. P*_i_* stands for the modified weighting of the *i*th individual index. With this step we deal with the extreme values and modify the weighting of each individual particulate matter pollutant. Accordingly, PAQI is calculated with Equation 3:

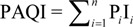
(3)


### 2.4. Per Capita and Population-Weighted PM_2.5_ Concentration Calculation

In this paper, per capita PM_2.5_ concentration (PC-PM_2.5_) and population-weighted PM_2.5_ concentration (PW-PM_2.5_) are calculated with Equations 4 and 5 based on grid computing to indicate population exposure to PM_2.5_. Both PC-PM_2.5_ and PW-PM_2.5_ are obtained from provincial statistics:

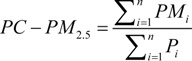
(4)

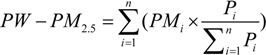
(5)
where, PM*_i_* is defined as the *i*th pixel value of PM_2.5_ concentration, P*_i_* is the *i*th pixel value of population density, *n* is the total pixel number of the certain province. PC-PM_2.5_ indicates the average exposure level of particulate matter of specific province, while PW-PM_2.5_ places emphasis on the actual effect of particulate matter to residents, taking population distribution into account. The weightiness of PM_2.5_ concentration in a dense population area is larger than in a suburb or sparse population area. The PW-PM_2.5_ method can indicate the PM_2.5_ exposure level of each resident every day. 

## 3. Results and Discussion

### 3.1. Spatial Distribution of PM Pollution

**Figure 2:**
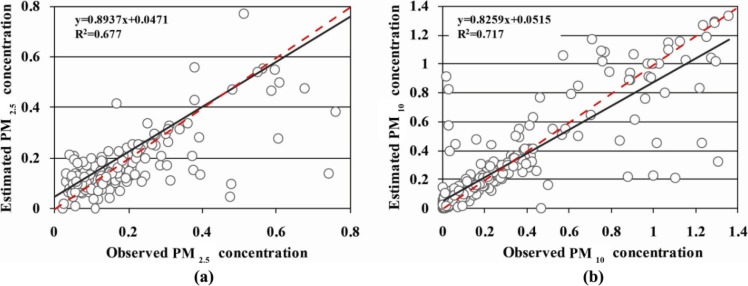
Accuracy of particulate estimation concentrations. (**a**) The accuracy of PM_2.5_ estimation; (**b**) The accuracy of PM_10_ estimation.

Comparing the estimation result with the PM observation data, the comparative analysis consists of two parts: accuracies and absolute percentage errors (APE) [19], the correlation coefficients of estimated PM_10_ and PM_2.5_ are respectively 0.85 and 0.82 (Figure 2), while the absolute percentage errors are about 29% and 25%. These results demonstrate the feasibility of the estimation model:

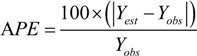
(6)
where, Y_est_ is the estimated concentration and Y_obs_ is the observed concentration in validation data set.

**Figure 3:**
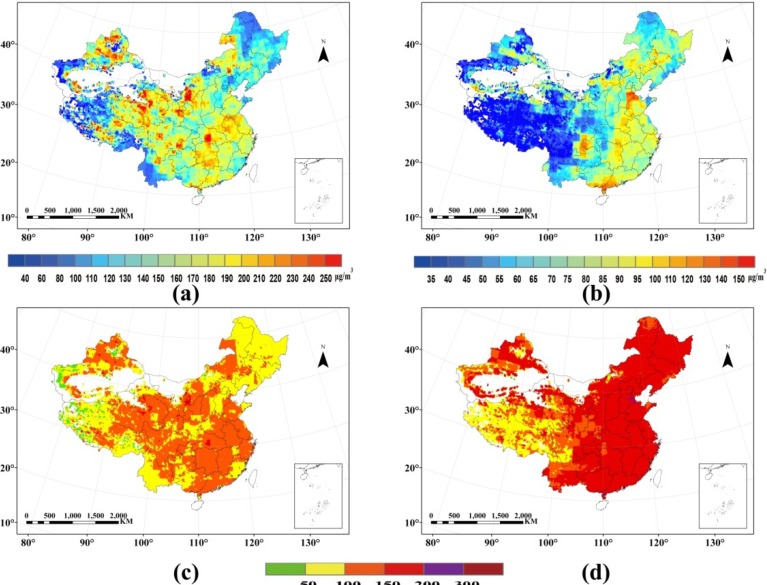
(**a**) Spatial distribution of PM_10_ concentration. (**b**) Spatial distribution of PM_2.5_ concentration. (**c**) Spatial distribution of IAQI_PM10_ concentration. (**d**) Spatial distribution of IAQI_PM2.5_ concentration.

The spatial distributions of particulate matter pollution over China 2010 are shown in Figure 3. The direct comparison between PM_10_ and PM_2.5_ concentration is meaningless due to the different threshold standards, hence IPM_10_ and IPM_2.5_ are calculated according to the Chinese AQI standard. Figure 3c,d reveal an obvious spatial differentiation. As to IPM_10_, it presents a good or light pollution state, while IPM_2.5_ shows a moderate pollution situation in the eastern China, and a particularly heavy pollution in Hebei Province. Overall, China suffered relatively more serious PM_2.5_ pollution than PM_10_ pollution in 2010.

**Figure 4:**
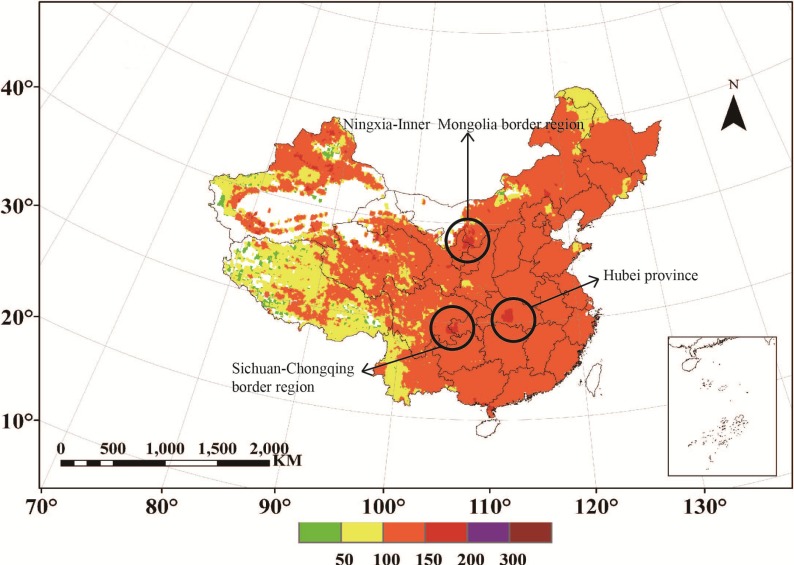
The spatial distribution of calculated PAQI over mainland China in 2010.

Owing to the spatial differentiation between IPM_10_ and IPM_2.5_, when evaluating particulate pollution level of a certain area or doing research on particulate health effects, both PM_10_ and PM_2.5_ should be taken into account at the same time. That’s why PAQI is needed. With the PAQI calculation method described in Section 3, the spatial distribution of PAQI over mainland China in 2010 is calculated (Figure 4). The classification criterion here for PAQI follows the Chinese AQI standard.

Compared with the two individual air quality indexes, PAQI shows an entirely different situation. PAQI over mainland China in 2010 is overall less than 200 (threshold of moderate pollution). There are three regions obviously in a moderate-pollution situation—Hubei Province, the Sichuan-Chongqing border region and the Ningxia-Inner Mongolia border region.

One possible explanation for this phenomenon is that the high value of Hubei Province appeared in Jingmen City and its neighboring region may be associated with coal burning and the construction dust; the Sichuan Basin is affected by the meteorological conditions, which are not conductive to the dilution of atmospheric particulate matter concentration; the high value in the Ningxia-Inner Mongolia border region (Shizuishan City, Wuhai City and Dengkou City, China) has a certain relation to the development of industry, it suffers from the most serious pollution in the Yellow River Basin because many mining industries are grouped on this region.

### 3.2. Population Exposure Assessment

**Figure 5:**
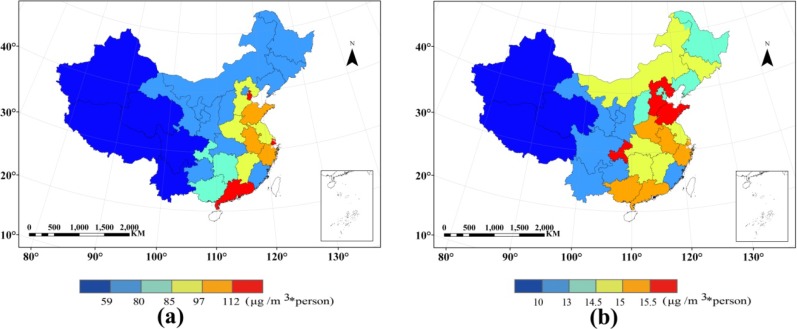
(**a**) Spatial distribution of per capita PM_2.5_ concentration (provincial statistical). (**b**) Spatial distribution of population-weighted PM_2.5_ concentration (provincial statistical).

PC-PM_2.5_ and PW-PM_2.5_ of China in 2010 are shown in Figure 5. The spatial distribution of the results is illustrated in the province-level. The distribution of PC-PM_2.5_ and PW-PM_2.5_ is apparently different. The high-level of PC-PM_2.5_ of China in 2010 is concentrated in the Eastern area. The provinces with highest PC-PM_2.5_ are Guangdong, Shanghai, and Tianjin. The 2nd highest places are Shandong, Anhui, and Zhejiang. The high-level areas of PW-PM_2.5_ are Hebei, Chongqing, and Shandong, and followed by Henan, Anhui, Zhejiang, Guangdong, and Guangxi. That is, PC-PM_2.5_ in Guangdong, Shanghai, and Tianjin is high in total, but the potential high pollution area is sparsely populated. Although PC-PM_2.5_ in Hebei and Chongqing is lower than in the high-level area, the population in the potential high pollution area is denser. Shandong Province is a special case, as it is not only located in the high-level concentration area, but also the population in the potential high pollution area is dense. The comparison between two indicators of PM_2.5_ population exposure level indicates the effect of population distribution on the regional PM_2.5_ exposure statics.

## 4. Conclusions

In this study, PM_10_ and PM_2.5_ concentrations of mainland China in 2010 are estimated with an ANN model, and then IPM_10_ and IPM_2.5_ are calculated based on grid computing, The spatial distribution of IPM_10_ demonstrates obvious differences with IPM_2.5_. IPM_10_ presents a good or light pollution state while IPM_2.5_ shows a moderate-pollution situation in eastern China, with a particularly heavily polluted region in Hebei Province. 

On the basis of the two individual indexes, we develop a new particulate air quality index (PAQI) based on a weighting method. It is shown that the PAQI over mainland China in 2010 shows overall a light pollution state, while some regions suffer a moderate-pollution situation.

As to the population exposure to PM_2.5_, the provinces with highest per capita PM_2.5_ concentration are Guangdong, Shanghai, and Tianjin, while the high-level areas of population-weighted PM_2.5_ concentration are Hebei, Chongqing, and Shandong. By comparison, the results demonstrate that the population-weighted PM_2.5_ concentration is more in accord with the actual situation and suitable for human health risk assessment. In future research, more attention can be paid to improving the spatial resolution of PAQI and discussion of its effects on human health.
